# Development of Novel Fractal Method for Characterizing the Distribution of Blood Flow in Multi-Scale Vascular Tree

**DOI:** 10.3389/fphys.2021.711247

**Published:** 2021-07-29

**Authors:** Peilun Li, Qing Pan, Sheng Jiang, Molei Yan, Jing Yan, Gangmin Ning

**Affiliations:** ^1^Department of Biomedical Engineering, Zhejiang University, Hangzhou, China; ^2^College of Information Engineering, Zhejiang University of Technology, Hangzhou, China; ^3^Department of Intensive Care Medicine, Zhejiang Hospital, Hangzhou, China

**Keywords:** multi-scale, vascular tree, fractal, vascular modeling, blood flow heterogeneity

## Abstract

Blood perfusion is an important index for the function of the cardiovascular system and it can be indicated by the blood flow distribution in the vascular tree. As the blood flow in a vascular tree varies in a large range of scales and fractal analysis owns the ability to describe multi-scale properties, it is reasonable to apply fractal analysis to depict the blood flow distribution. The objective of this study is to establish fractal methods for analyzing the blood flow distribution which can be applied to real vascular trees. For this purpose, the modified methods in fractal geometry were applied and a special strategy was raised to make sure that these methods are applicable to an arbitrary vascular tree. The validation of the proposed methods on real arterial trees verified the ability of the produced parameters (fractal dimension and multifractal spectrum) in distinguishing the blood flow distribution under different physiological states. Furthermore, the physiological significance of the fractal parameters was investigated in two situations. For the first situation, the vascular tree was set as a perfect binary tree and the blood flow distribution was adjusted by the split ratio. As the split ratio of the vascular tree decreases, the fractal dimension decreases and the multifractal spectrum expands. The results indicate that both fractal parameters can quantify the degree of blood flow heterogeneity. While for the second situation, artificial vascular trees with different structures were constructed and the hemodynamics in these vascular trees was simulated. The results suggest that both the vascular structure and the blood flow distribution affect the fractal parameters for blood flow. The fractal dimension declares the integrated information about the heterogeneity of vascular structure and blood flow distribution. In contrast, the multifractal spectrum identifies the heterogeneity features in blood flow distribution or vascular structure by its width and height. The results verified that the proposed methods are capable of depicting the multi-scale features of the blood flow distribution in the vascular tree and further are potential for investigating vascular physiology.

## Introduction

The microcirculation is the end destination of the cardiovascular system and the patency of microvascular perfusion is essential for the maintenance of tissue metabolism (Ince, 2005; Guven et al., 2020). Various cardiovascular diseases influence the blood perfusion and thus impair the physiological function of organs (Efimova et al., 2008; Kitagawa et al., 2009; Alosco et al., 2013, 2014). These findings imply that the blood perfusion may act as an important index for the physiological states of living bodies. The blood perfusion can be indicated by the blood flow distribution in the vascular tree. The blood flow in a vascular tree is distributed at different generations, varying in a large range of scales, and the blood flow distribution at a certain generation is directly affected by the superior generation. In the meantime, there is a huge difference between the magnitude of the blood flow at different generations. However, the conventional statistical parameters for characterizing the blood flow distribution, like the coefficient of variation (CV) (Bassingthwaighte et al., 2001; Pries and Secomb, 2009), ignored the connection of the blood flow among multiple scales. To develop a unified description of the blood flow distribution covering all scales remains a big challenge.

To depict the scale-independent characteristic of objects, the fractal theory provides an efficient approach for multi-scale analysis (Mandelbrot, 1982). Presently, a few studies have made an effort on investigating the fractal characteristics of blood flow distribution in the vascular tree directly or indirectly. Van Beek et al. (1989) uncovered the fractality of the relative dispersion of blood flow distribution. Zamir (2001) defined the fractal dimension based on the relationship between the vessel diameter and blood flow according to Murray’s law. Grasman et al. (2003) described that the distribution of blood flow at the same generation is multifractal. In all these studies, the unified description for a vascular tree by the fractal parameter all demands that the vascular tree should be a perfect binary tree, in which all interior branch nodes have two daughter branches and all terminals have the same depth or generation. However, the structures of real vascular trees are diverse which limits the physiological application of the methods above.

The fractal analysis has been widely used to investigate the geometrical characteristics of the vasculatures (Cheng and Huang, 2003; Stosic and Stosic, 2006; Lorthois and Cassot, 2010; Gould et al., 2011; Nadal et al., 2020). It inspires us to introduce the established fractal methods for geometrical architecture analysis into the hemodynamic study, and further develop a universal fractal depiction for blood flow distribution. The conservation law is common during the emergence of fractal and multifractal (Hassan, 2019). In fractal geometry, this law presents as the conservation of the number of signal pixels in an image. On the other hand, the total volume of blood flow in the vascular tree also obeys the conservation law. This consistency makes it possible to apply the principle of the fractal method for geometry to the analysis of blood flow by appropriate modification.

In this study, the primary aim is to establish fractal methods for analyzing the blood flow distribution which is potential to be applied to real vascular trees. To achieve this goal, we firstly modified the fractal methods in geometry to accommodate the situation of blood flow and then applied the established methods on experimental data to test the validity. Further, to explore the physiological significance of the yielded fractal parameters, the blood flow distribution in vascular trees with fixed structure or with varying structures were examined in which the hemodynamics was simulated based on a hemodynamic model (Yang and Wang, 2013) and a rheological model (Pries and Secomb, 2005).

## Materials and Methods

### Establishment of Fractal Methods for Blood Flow

The fractal dimension is the most important parameter to quantify the fractality of objects. And measuring the information dimension is an efficient way to estimate the fractal dimension in geometry (Pitsianis et al., 1989; Liu et al., 2018). For the calculation of information dimension, non-overlapping boxes are adopted to cover the image of the object and the mass probability of each box, which is defined as the ratio of the number of signal pixels in the box to that of the whole image, is obtained. And the information dimension *D*_*I*_ (Pitsianis et al., 1989) is estimated as:

(1) $$D_{I}=\lim_{L\to 0}\frac{\sum_{i=1}^{N\,\left(L\right)}-P_{i}\,\log P_{i}}{\log\left(1/L\right)}$$

where $\sum_{i=1}^{N\,\left(L\right)}-P_{i}\,\log P_{i}$ is the total entropy of mass according to the information theory, *P*_*i*_ is the mass probability of the *i*th box and *N*(*L*) is the number of boxes needed to cover the image with size *L*.

**FIGURE 1:**
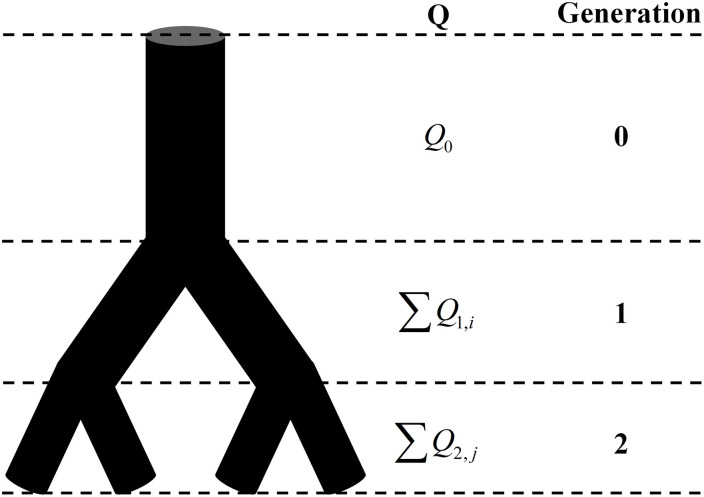
The schematic of the conservation of flow at different generations. Q is the blood flow and *Q*_0_ = ∑*Q*_1,*i*_ = ∑*Q*_2,*j*_.

The total mass, which is the number of signal pixels in the whole image, obeys the law of conservation regardless of the box size. And so does the blood flow. As shown in Figure 1, the total volume of blood flow at the same generation also follows the law of conservation no matter how many times the vascular tree bifurcates. Thus, with appropriate modification, the fractal methods in fractal geometry can be introduced to investigate the fractality of blood flow. By replacing the mass probability *P*_*i*_ in Eq. 1 with the flow probability *p*_*i*_ and 1/*L* with the number of vessel segments *N*(*g*) at generation *g*, the expression of the fractal dimension for the blood flow *D*_*Q*_ is derived as:

(2) $$D_{Q}=\lim_{N\,\left(g\right)\to \infty }\frac{\sum -p_{i}\,\log p_{i}}{\log N\,\left(g\right)}$$

Practically, *D*_*Q*_ is estimated as the slope of the linear fitting curve of the discrete data pair (*log*⁡*N*(*g*), ∑−*p*_*i*_*log*⁡*p*_*i*_) (Wang et al., 2019). The precondition of the fractal analysis is scale invariance. And the strong linearity over three orders of magnitude of the fitting curve can be taken as the criteria of the existence of scale invariance (Halley et al., 2004).

For the fractal analysis in geometry, the box-counting dimension (So et al., 2017; Nayak et al., 2019), also known as capacity dimension, is the most popular. This method also requires the image to be covered by non-overlapping boxes. However, the box-counting dimension method only considers the existence of signal pixels in the box but ignores the number of pixels. When applied to blood flow analysis, it produces fractal dimension about the vascular structure other than the distribution of blood flow. In contrast, the information dimension method takes the quantity of blood flow into account and thus can reflect the blood flow distribution.

Very few objects possess perfect mono-fractality exhibiting a single fractal dimension (Gould et al., 2011). In reality, objects with the subsets having different scaling properties are much more common and the estimation of multifractality is more desirable. For the multifractal measure of blood flow, the multifractal spectrum *f*(α)∼α of the blood flow is adopted (Chhabra and Jensen, 1989) and modified as:

(3) $$f\,\left(q\right)=\lim_{N\,\left(g\right)\to \infty }\frac{\sum \mu_{i}\,\left(q\right)\,\log\left[\mu_{i}\,\left(q\right)\right]}{\log N\,\left(g\right)}$$

(4) $$\alpha \,\left(q\right)=\lim_{N\,\left(g\right)\to \infty }\frac{\sum \mu_{i}\,\left(q\right)\,\log p_{i}}{\log N\,\left(g\right)}$$

in which

(5) $$\mu_{i}\,\left(q\right)=\frac{p_{i}^{q}}{\sum p_{i}^{q}}$$

where *q* is the moment order. And the range of the spectrum Δα = α_*max*_ − α_*min*_ can be used to measure the degree of multifractality (Halsey et al., 1986).

### Generalization of the Established Methods

**FIGURE 2:**
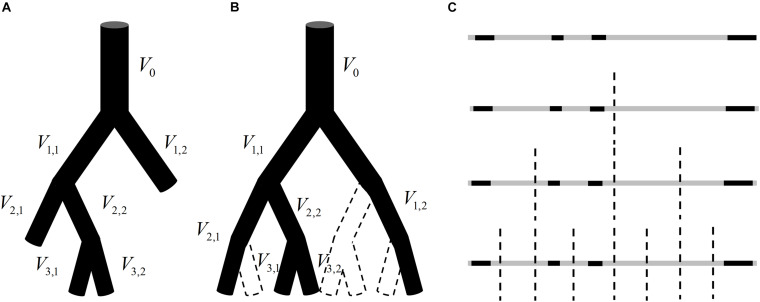
**(A)** An instance of a vascular tree with the maximal generation of 3. **(B)** A hypothetical perfect binary tree for the vascular tree in panel **(A)** and there is no blood flow in the vessel segment surrounded by dotted lines. **(C)** The analogy of the blood flow distribution in panel **(B)** to a one-dimension graph.

The methods given in Section “Establishment of Fractal Methods for Blood Flow” are based on the premise that the total blood flow at the same generation in a vascular tree obeys the law of conservation. This premise is valid for a perfect binary tree, as shown in Figure 1, but not for real vascular trees as shown in Figure 2A. If the branch which stops bifurcating before reaching the maximal generation is regarded as a branch covering multiple generations, the vascular tree in Figure 2A can be thought of as the perfect binary tree as shown in Figure 2B. In this case, the proposed methods can be applied and a strategy for the calculation is raised: ***If a vessel segment stops bifurcating at generation n* (*n is smaller than the maximal generation of vascular tree*), *it will be involved in the calculation at all the generations greater than n*.**

That is to say, for the vascular tree in Figure 2A, *V*_2,1_ will be included in the calculation of total entropy at generation 3 and *V*_1,2_ will be included in the calculation at both generation 2 and generation 3. It should be noted that the number of vessel segments *N* in Eq. 2 should be 2*^*n*^* at generation *n* but not the number of vessel segments at this generation. The reason is explained below.

The blood flow distribution in the vascular tree in Figure 2B can be analogous to a one-dimension graph as shown in Figure 2C. The gray segments indicate the range of the graph and the black segments reflect the blood flow in vessel segments. Each bisection corresponds to a bifurcation of the vascular tree and divides this geometrical structure into smaller subsections. When adopting Eq. 1 for the estimation of fractal dimension for this one-dimension graph, *L* is corresponding to the length of the smallest subsection. And each bisection of the graph halves *L* no matter whether there is always a black segment in each subsection. The number of subsections should be 2*^*n*^* after *n* bisections. Similarly, the number of vessel segments *N*(*g*) in Eq. 2 should be 2*^*n*^* at generation *n.*

### Vascular Tree Construction and Hemodynamic Simulation

By now, the methods established in “Establishment of Fractal Methods for Blood Flow” and “Generalization of the Established Methods” can be used to characterize the blood flow distribution in arbitrary vascular trees. For validation, the established fractal methods were tested in a real arterial tree (Reglin et al., 2009, 2017; Wang et al., 2019) under normal and ischemic state. Besides, the capability of the derived fractal parameters was examined in two situations.

For the first situation, the vascular tree was fixed to be a perfect binary tree and the blood flow distribution was adjusted by the split ratio. For a bifurcation with a parent vessel segment and two daughter branches, the split ratio *r* is defined as the ratio of the smaller blood flow to the larger one in the two daughter branches, ranging from 0 to 1. Assuming that *r* is constant throughout the perfect binary tree, the fractal dimension for blood flow can be obtained as shown in Eq. 6. The detailed derivation of the equation is given in the Appendix.

(6) $$D_{Q}\,\left(r\right)=\frac{\left(1+r\right)\,\log\left(1+r\right)-r\,\log r}{\log2\cdot \left(1+r\right)}$$

For the second situation, a series of vascular trees were constructed. The structures of these vascular trees were diverse while the blood flow distribution was estimated under the same boundary condition.

The successive dichotomous division is the most common branching pattern of the vascular tree, in which a parent vessel segment is divided into two daughter branches (Zamir, 2001). Based on this pattern, the construction of a vascular tree calls for the determination of the vessel diameter and length. For a bifurcation with the diameter of the parent vessel being *d*_0_ and those of the two daughter branches being *d*_1_ and *d*_2_, a power-law relationship between the diameters is given as shown in Eq. 7 according to Murray’s law.

(7) $$\begin{array}{c} \left\{ \begin{array}{c} d_{0}^{k}=d_{1}^{k}+d_{2}^{k}\\ \lambda =\frac{d_{2}}{d_{1}} \end{array}\right. \end{array}$$

where *d*_0_ > *d*_1_ ≥ *d*_2_, *k* is bifurcation exponent and λ is asymmetry ratio. It is reported that the *k* value varies from 2.33 to 3.0 (Gabrys et al., 2005). And a value above 0.6 is most commonly observed for λ (Schmidt et al., 2004; Cheung et al., 2011; Takahashi, 2014). Based on the power-law relationship, the diameters of all vessel segments in a vascular tree can be estimated with the given root diameter and cut-off diameter. In this study, the root diameters of all constructed vascular trees are set as 300 μm and all the terminal diameters are 10 μm, the size of capillaries. An empirical formula (Takahashi et al., 2009) is adopted to obtain the length *l* (μm) from the diameter *d* (μm):

(8) $$l=7.4\cdot {\left(\frac{d}{2}\right)}^{1.15}$$

With the estimated vessel diameters and lengths, a vascular tree can be constructed, and serves for the hemodynamic simulation. According to Hagen-Poiseuille’s law as shown in Eq. 9, the blood flow *Q* (μm^3^/s) in a vessel segment is proportional to the pressure drop Δ*P* (Pa) between the inlet and outlet. And the flow resistance *R* is determined by vessel diameter *d* (μm), vessel length *l* (μm), and blood viscosity μ (Pa ⋅ s).

(9) $$\begin{array}{c} \left\{ \begin{array}{c} Q=\frac{△\,P}{R}\\ R=\frac{128\,\mu \,l}{\pi \,d^{4}} \end{array}\right. \end{array}$$

Fahraeus and Lindqvist (1931) reported a decline in apparent blood viscosity with decreasing tube diameter, the so-called Fahraeus-Lindqvist effect. Among the models to describe the relationship between the blood viscosity μ (mPa ⋅ s) and the diameter of the vessel segment *d* (μm), the model proposed by Pries and Secomb (2005) matches well with the *in vivo* experimental data. In this model, the *in vitro* viscosity is firstly estimated as:

(10) $$\mu_{v\,i\,t\,r\,o}=1+\left(\mu_{0.45}-1\right)\cdot \frac{{\left(1-H_{d}\right)}^{C}-1}{{\left(1-0.45\right)}^{C}-1}$$

in which *H*_*d*_ (%) is the hematocrit and:

(11) $$\mu_{0.45}=220\,e^{-1.3\,d}+3.2-2.44\,e^{-0.06\,d^{0.645}}$$

(12) $$C=\left(0.8+e^{-0.075\,d}\right)\cdot \left(\frac{1}{1+10^{-11}\cdot d^{12}}-1\right)+\frac{1}{1+10^{-11}\cdot d^{12}}$$

The estimation of the *in vivo* viscosity should take account of the effect of the endothelial surface layer. This will involve two parameters, the effective diameter *d*_*eff*_ = d − *2W*_*eff*_ and the physical diameter *d*_*ph*_ = d − *2W*_*ph*_. The effective thickness of the layer *W*_*eff*_ and physical thickness of the layer *W*_*ph*_ are estimated as:

(13) $$W_{e\,f\,f}=W_{a\,s}+W_{p\,e\,a\,k}\,\left(1+H_{d}\cdot E_{H\,D}\right)$$

(14) $$W_{p\,h}=W_{a\,s}+W_{p\,e\,a\,k}\cdot E_{p\,e\,a\,k}$$

(15) $$W_{a\,s}=\left\{ \begin{array}{cc} 0\,\;\;\;\;\;\;\;d\leq d_{o\,f\,f}\; & \\ \frac{d-d_{o\,f\,f}}{d+d_{50}-2\,d_{o\,f\,f}}\cdot W_{m\,a\,x}\,d\geq d_{o\,f\,f}\; & \end{array}\right.$$

(16) $$W_{a\,s}=\left\{ \begin{array}{cc} 0\,\;\;\;\;\;\;\;\;d\leq d_{o\,f\,f}\; & \\ E_{a\,m\,p}\cdot \frac{d-d_{o\,f\,f}}{d_{c\,r\,i\,t}-d_{o\,f\,f}}\,\;\;\;d_{o\,f\,f}<d\leq d_{c\,r\,i\,t}\; & \\ E_{a\,m\,p}\cdot e^{-E_{w\,i\,d\,t\,h}\cdot \left(d-d_{c\,r\,i\,t}\right)}\;d>d_{c\,r\,i\,t}\; & \end{array}\right.$$

Based on the experimental data, *E*_*HD*_ = 1.18, *E*_*peak*_ = 0.6, *E*_*amp*_ = 1.1, *E*_*width*_ = 0.03, *D*_*off*_ = 2.4 μm, *D*_*crit*_ = 10.5 μm, *D*_50_ = 100 μm, and *W*_*max*_ = 2.6μm. By replacing the *d* in Eqs 11 and 12 with *d*_*ph*_, we can get the *in vitro* viscosity μ_*vitro*_. And the *in vivo* viscosity μ_*vivo*_ can be obtained as below.

(17) $$\mu_{v\,i\,v\,o}=\mu_{v\,i\,t\,r\,o}\cdot {\left(\frac{d}{d_{e\,f\,f}}\right)}^{4}$$

**FIGURE 3:**
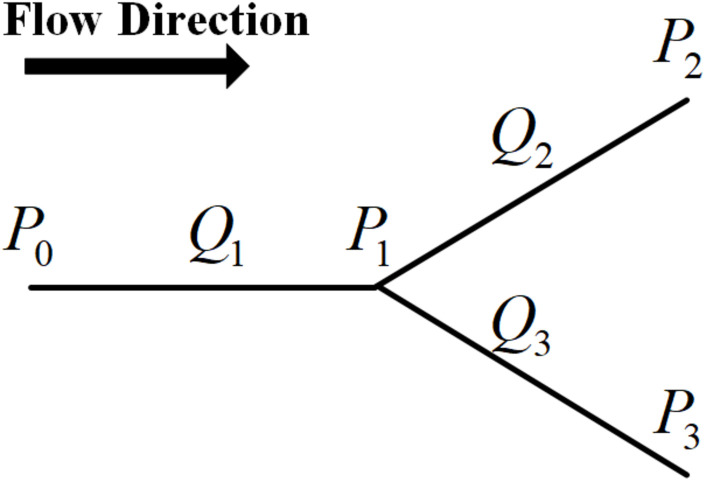
The schematic of the hemodynamic parameters at a bifurcation.

For the bifurcation as shown in Figure 3, the relationship between the hemodynamic parameters can be given by Eq. 18 based on Hagen-Poiseuille’s law and the conservation law of flow.

(18) $$\frac{P_{0}-P_{1}}{R_{1}}+\frac{P_{2}-P_{1}}{R_{2}}+\frac{P_{3}-P_{1}}{R_{3}}=0$$

Without losing generality, in the hemodynamic simulation we prescribed the inlet pressure as 1 mmHg and the outlet pressure at all terminal branches as 0 mmHg (Yang and Wang, 2013). With each bifurcation of the vascular tree assigned an equation like Eq. 18, the blood pressure at each branch node can be obtained by solving these equations. Further, the blood flow in each vessel segment is estimated by Eq. 9 and finally the blood flow distribution in a tree can be acquired.

### Numerical Solution

In this study, all the calculations and simulations were programmed by MATLAB R2019a (MathWorks Co., MA, United States). Firstly, the node information for each constructed vascular tree was obtained. Then, the blood flow in each vessel segment of the constructed vascular tree was captured by solving the equations set. Ultimately, the fractal dimension and multifractal spectrum were calculated. All the results about the fractal parameters were presented as Mean ± SD.

## Results

### The Validation of the Proposed Methods

**FIGURE 4:**
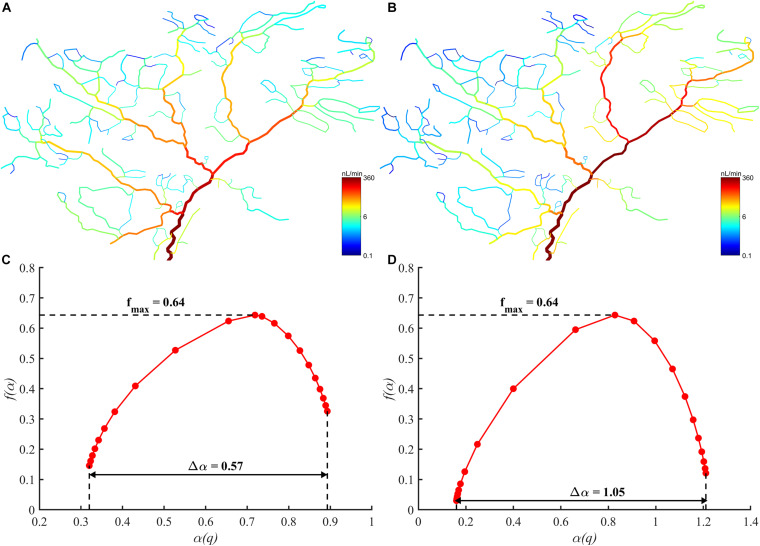
**(A)** The blood flow distribution in the arterial tree under normal state. **(B)** The blood flow distribution in the arterial tree under ischemic state. **(C)** The multifractal spectrum of the blood flow distribution under normal state. **(D)** The multifractal spectrum of the blood flow distribution under ischemic state.

To test the validity of the proposed methods, the fractal, and multifractal analysis were conducted on a real arterial tree under normal and ischemic state. The blood flow distribution in these two states is as shown in Figures 4A,B. The fractal dimension for the normal and ischemic state are 0.53 and 0.40, respectively. As for the multifractal spectrum for the blood flow, the results are shown in Figures 4C,D. We can observe that the multifractal spectrums for both states appear as curves indicating the existence of multifractality. While the maximal values of the multifractal spectrum for the two states are the same, the range of the multifractal spectrum Δα for the ischemic state is wider than that for the normal state which indicates a higher degree of multifractality for the ischemic state. The results verified the ability of the proposed methods in distinguishing different blood flow distribution in a real vascular tree.

### The Fractality of Blood Flow in the Perfect Binary Vascular Tree

**FIGURE 5:**
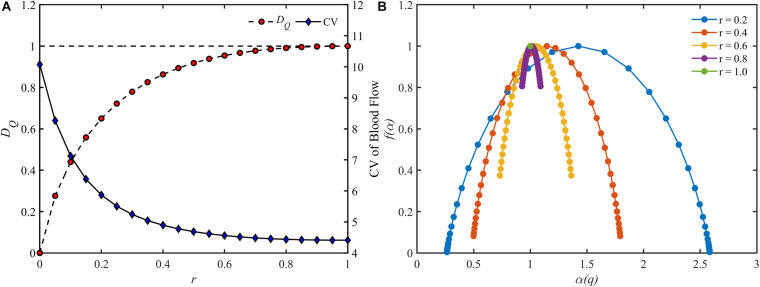
The change of different parameters of the blood flow for different split ratios in a perfect binary vascular tree. **(A)** The fractal dimension and the coefficient of variation. **(B)** The multifractal spectrum.

The blood flow distribution in the perfect binary vascular tree was evaluated by both the fractal parameters and the CV, which is defined as the standard deviation divided by the mean value. For a perfect binary vascular tree with the identical split ratio for each bifurcation, the fractal dimension for blood flow was obtained based on Eq. 6. Figure 5A shows the trends of fractal dimension and the coefficient of variation with the change of split ratio. It is noticed that the fractal dimension increases monotonically from 0 to 1 with the increment of the split ratio. In the case *r* = 1, the distribution of blood flow has the highest value of fractal dimension. And the CV decreases from 10.1 to 4.4 with the increment of the split ratio.

As for the multifractal characteristic, the multifractal spectrums of the blood flow are shown in Figure 5B. With the decrement of the split ratio, the range of the multifractal spectrum Δα expands while the maximal value remains unchanged. It is worth noting that the multifractal spectrum is presented as a point when *r* = 1, implying the absence of multifractality.

**FIGURE 6:**
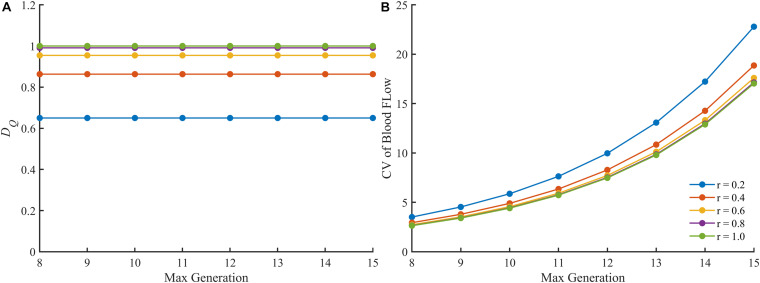
The change of different parameters of the blood flow in the perfect binary vascular trees with different maximal generations. **(A)** The fractal dimension. **(B)** The coefficient of variation.

We also examined the fractal dimension and the CV of the blood flow in perfect binary vascular trees with different maximal generations. As shown in Figure 6, the fractal dimension holds steady with the change of maximal generation while the CV varies greatly.

### The Fractality of Blood Flow in Constructed Vascular Trees

**FIGURE 7:**
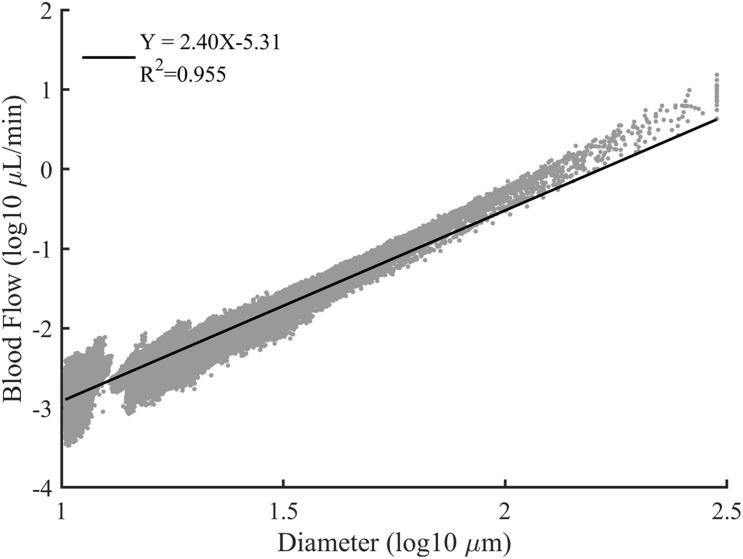
The log-log plot of the blood flow rate versus the vessel diameter for all constructed vascular trees. The solid line is the best fit result of linear regression.

Vascular trees with diverse structures were constructed. To reflect the heterogeneity in the real vascular tree, the bifurcation exponent *k*, and asymmetry ratio λ of the bifurcations in each constructed vascular tree were set following the normal distribution. And ten vascular trees were constructed for each pair of *k* and λ. The statistical characteristics of these vascular trees are shown in Table 1. In these constructed vascular trees, the hemodynamics was simulated and the results are shown in Figure 7. We can see that there exists a strong linear relationship between the logarithmic values of the diameter and the blood flow rate.

**TABLE 1:** The characteristics of the constructed vascular trees.

*k*	λ	Vessel number	Max generation	*k*	λ	Vessel number	Max generation
2.7	0.60	7471 ± 97	33.8 ± 1.7	2.3	0.80	2822 ± 334	15.3 ± 0.6
	0.65	8261 ± 130	28.2 ± 1.2	2.4		3525 ± 281	15.8 ± 0.6
	0.70	9012 ± 83	24.9 ± 0.8	2.5		5102 ± 469	16.7 ± 0.5
	0.75	9777 ± 73	21.5 ± 0.7	2.6		6987 ± 887	17.4 ± 0.7
	0.80	10692 ± 99	19.5 ± 0.7	2.7		10210 ± 1389	18.8 ± 0.9
	0.85	11387 ± 112	17.8 ± 0.4	2.8		14672 ± 1038	20.2 ± 0.7
	0.90	12189 ± 93	16.4 ± 0.5	2.9		19149 ± 2544	21.2 ± 0.4
	0.95	12818 ± 110	15.3 ± 0.5	3.0		27222 ± 1774	22.2 ± 0.9
	1.00	16234 ± 102	13.0 ± 0.0				

**FIGURE 8:**
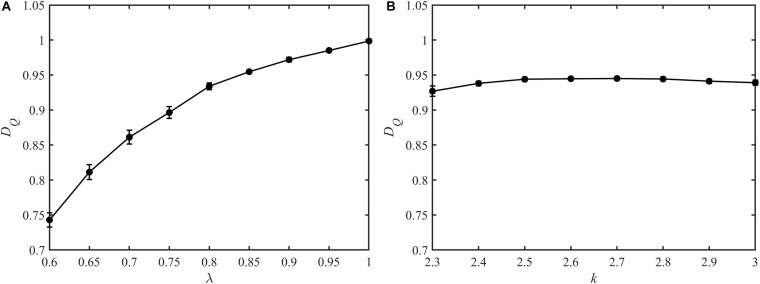
The fractal dimension for blood flow of the vascular trees with varying λ **(A)** and *k* **(B)**.

The fractal dimensions for blood flow in the constructed vascular trees are shown in Figure 8. For the vascular trees with the mean *k* value of 2.7, the fractal dimension increases monotonically from 0.74 ± 0.01 to 1.00 ± 0.00 with λ rising from 0.60 to 1. While for the vascular trees with the mean λ value of 0.8, the fractal dimension fluctuates between 0.91 and 0.95 with the increment of *k*.

**FIGURE 9:**
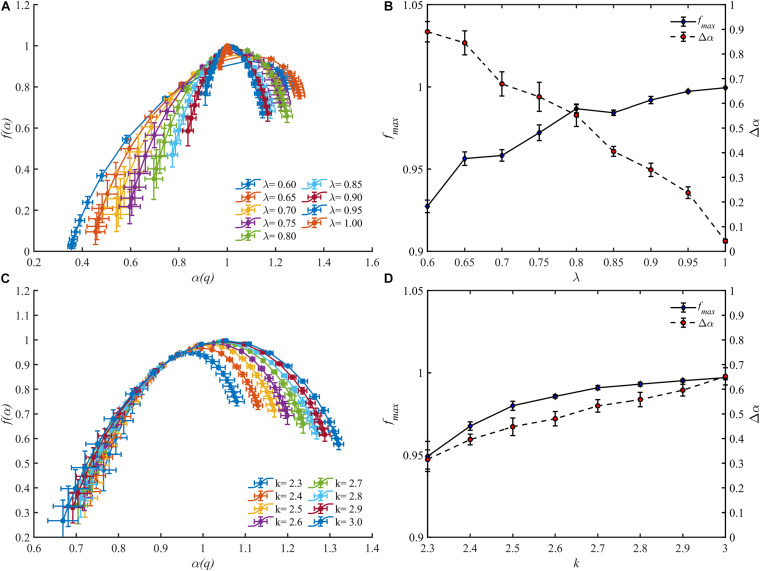
The multifractal spectrums for blood flow of the vascular trees with varying λ **(A)** and *k* **(C)**. The range Δα and the maximal value *f*_*max*_ of the multifractal spectrums with varying λ **(B)** and *k* **(D)**.

The multifractal spectrums of the blood flow for these vascular trees are presented in Figures 9A,C. It is observed that the multifractality of blood flow exists in all vascular trees but the multifractal spectrums fluctuate. With the increment of λ, the range of the multifractal spectrum Δα narrows from 0.89 ± 0.04 to 0.04 ± 0.01 and the maximal value grows from 0.93 ± 0.01 to 1.00 ± 0.00. And with the increment of *k*, Δα expands from 0.32 ± 0.04 to 0.65 ± 0.03 and the maximal value grows from 0.95 ± 0.01 to 0.99 ± 0.00. The features of the multifractal spectrums are shown in Figures 9B,D. Compared with the fractal dimensions, the difference of multifractal spectrums among these vascular trees is more striking.

## Discussion

### Validity of the Hemodynamic Simulation

The hemodynamic simulation was conducted to investigate the variation of fractal parameters with varying blood flow distribution. To make sure that the obtained blood flow distribution is reasonable, a quantitative comparison of the hemodynamic simulation with the existing physiological studies is necessary. For avoiding losing the generality, the boundary condition in the present work was prescribed with an inlet pressure of 1 mmHg and an outlet pressure of 0 mmHg. However, the pressure drop between the inlet and outlet may vary in different studies. Thus, for quantitative comparison, it is more appropriate to examine the relative indices.

As shown in Figure 7, a strong linear relationship exists between the logarithmic values of the diameter and the blood flow rate. This is consistent with the assertion in Huo and Kassab (2016) that there is a scaling law between the blood flow rate and diameter. The slope of the fitting line indicates the relation between the blood flow rate and vascular diameter thus can be an indicator for quantitative comparison. Within a similar diameter range, the slope with a value of 2.40 produced in our work is comparable with the reported work of 1.97 (Wang et al., 2009), 2.0 ± 0.2 (Pijewska et al., 2020), 2.33 (Huo and Kassab, 2012), and 2.49 ± 0.09 (Haindl et al., 2016). Thus, it can be concluded that the simulation results of our work are reasonable.

### Physiological Significance of the Fractal Parameters

Two fractal parameters, i.e., fractal dimension and multifractal spectrum, were obtained in this study to investigate the fractality and multifractality of blood flow.

By definition, the fractal dimension is determined by the total entropy of blood flow. And the total entropy is calculated by considering the existence as well as the quantity of blood flow in the vessel segment. The existence and the quantity of blood flow are corresponding to the vascular structure and the blood flow distribution, respectively. Thus, the fractal dimension characterizes the combination of the features of vascular structure and blood flow distribution. When the vascular structure is fixed, the lower entropy is obtained from the more heterogeneous distribution according to the information theory. That is to say, the fractal dimension reflects the degree of the blood flow heterogeneity for a specific vascular tree and the lower fractal dimension comes from the blood flow distribution with a higher degree of heterogeneity. The results in Figure 5A that the lower fractal dimension is corresponding to the lower split ratio also support this conclusion.

For a fractal object, the multifractal spectrum describes the scaling properties in different subsets. And when multifractality presents, the subsets of this object will be scaled by different multiples at the same *q* order moment during the calculation of the multifractal spectrum. Therefore, the degree of multifractality, which is measured by the width of the multifractal spectrum, essentially describes the degree of heterogeneity within the fractal object and it rises with the increment of the blood flow heterogeneity. This judgment is consistent with the results as shown in Figure 5B. The multifractal spectrum *f*(α(*q*)) reaches its maximal value when *q* = 0. In this case, the quantity of the blood flow volume no longer has an effect on the value of *f*(α(0)). In other words, the height of the multifractal spectrum reflects the heterogeneity or asymmetry of the vascular structure. The higher the multifractal spectrum the closer the vascular tree is to the perfect binary tree. And this makes clear why the height of the multifractal spectrums in Figures 4C,D or Figure 5B is the same. By means of the width and height, the multifractal spectrum separates the information about the vascular structure and blood flow distribution. In this sense, the multifractal spectrum makes the evaluation of the blood flow distribution in different vascular trees possible. As shown in Figure 9D, the heterogeneity of the vascular structure decreases with the increment of the bifurcation exponent while the heterogeneity of the blood flow distribution increases. The interaction of these two opposite trends may explain why the fractal dimension changes slightly with the bifurcation exponent as shown in Figure 8B.

Both the fractal dimension and the multifractal spectrum reflect the blood flow heterogeneity. Physiologically speaking, the change of blood flow heterogeneity is usually associated with pathological conditions. For microcirculation, the increment of blood flow heterogeneity can be an early indicator of diseases, such as sepsis and shock (Ince, 2005; Dubin et al., 2018; Ince et al., 2018) as well as peripheral vascular disease (Butcher et al., 2013). And the increase of blood flow heterogeneity can be depicted by the decrease of fractal dimension and the broadening of the multifractal spectrum. The multifractal spectrum can separate the information about the vascular structure and blood flow distribution. Thus, the multifractal spectrum is also able to distinguish the causes responsible for the change in blood flow heterogeneity, either by hemodynamic problems or by structural alteration due to the diseases such as large vessel stenosis (Kharche et al., 2018).

There are also some other quantitative or semi-quantitative methods for characterizing the blood flow heterogeneity (Bassingthwaighte et al., 1989; Pries and Secomb, 2009; Ince et al., 2018). In this study, the blood flow heterogeneity is also evaluated by CV. The results in Figure 5A confirm the availability of this statistical parameter. However, the CV treats different vessel segments in a vascular tree as independent components ignoring the connection of blood flow along the whole tree. This would make this parameter less accurate in some cases as discussed below. When the split ratio for each bifurcation in the perfect binary vascular tree is 1, the blood flow is evenly distributed for each generation in the vascular tree. The degree of blood flow heterogeneity should remain unchanged no matter what the maximal generation of the vascular tree is. And this property holds for the other values of the split ratio. As shown in Figure 6A, the fractal dimension stays the same with the change of the maximal generation. However, the values of CV for the vascular trees with different maximal generations are quite different. Considering both the connection and difference of blood flow in different vessel segments, the fractal parameters can provide a more accurate description of the blood flow heterogeneity for the tree-like vasculature.

### Limitations

It should be pointed out the hemodynamic simulation in this study was simplified. Nowadays, the RCL model has been developed for hemodynamic simulation in which the resistance (R), capacitance (C), and inductance (L) elements were used to mimic the effects of vessel resistance, vessel compliance, and blood inertia, respectively (Muller and Toro, 2014; Zhang et al., 2014). And models from 0D to 3D were established (Arciero et al., 2017; Liu et al., 2020). In the present hemodynamic model of microcirculation, only the resistance element for a vessel segment was considered. Although in the microcirculation the resistance element plays a dominant role in hemodynamics (Katanov et al., 2015; Nichols et al., 2015; Secomb, 2017) and the results show that the model is sufficient for produce varying blood flow distribution in a tree, a comprehensive model is worth being introduced in the future study.

## Conclusion

In this study, the fractal methods were introduced, with appropriate modification, to characterize the multi-scale properties of blood flow. The application of the methods to the real physiological data verified its ability in distinguishing the variety of blood flow distribution. The yielded parameters, as the fractal dimension and the multifractal spectrum for blood flow, can quantify the degree of blood flow heterogeneity. With the increase of blood flow heterogeneity, the fractal dimension decreases and the multifractal spectrum expands. And the investigation on various constructed vascular trees suggests that both the vascular structure and the blood flow distribution influence the fractal parameters. With the aid of the fractal dimension, it is possible to look into the change of blood flow heterogeneity in a specific vascular tree. While the multifractal spectrum can be utilized to assess the blood flow heterogeneity for different vascular trees by considering the blood flow distribution and the structure of vascular trees separately. It can be concluded that the proposed methods provide efficient tools to describe the multi-scale properties of the blood flow distribution and has the potential to assist the study of multi-scale vascular physiology.

## Data Availability Statement

The original contributions presented in the study are included in the article/supplementary material, further inquiries can be directed to the corresponding authors.

## Author Contributions

PL and QP: conceptualization and methodology. PL: algorithm and writing (original draft). SJ and GN: writing (review and editing). MY and JY: discussion of the results and their relevance. GN and JY: supervision and project administration. All authors approved the manuscript.

## Conflict of Interest

The authors declare that the research was conducted in the absence of any commercial or financial relationships that could be construed as a potential conflict of interest.

## Publisher’s Note

All claims expressed in this article are solely those of the authors and do not necessarily represent those of their affiliated organizations, or those of the publisher, the editors and the reviewers. Any product that may be evaluated in this article, or claim that may be made by its manufacturer, is not guaranteed or endorsed by the publisher.

## Appendix

For the perfect binary vascular tree where the split ratio of each bifurcation is identical, if we normalize the blood flow in the main vessel at generation 0 as 1 and denote the split ratio by *r*, there will be $C_{n}^{k}$ vessels with blood flow ${\left(\frac{r}{1+r}\right)}^{k}\,{\left(\frac{1}{1+r}\right)}^{n-k}$ at generation *n*. And the summed entropy of the blood flow at generation *n* is as below:

$$\sum_{k=0}^{n}C_{n}^{k}\,\left[-\frac{r^{k}}{{\left(1+r\right)}^{k}}\,\frac{1}{{\left(1+r\right)}^{n-k}}\,\log\left(\frac{r^{k}}{{\left(1+r\right)}^{k}}\,\frac{1}{{\left(1+r\right)}^{n-k}}\right)\right]$$

$$\;=-\sum_{k=0}^{n}C_{n}^{k}\,\left[\frac{r^{k}}{{\left(1+r\right)}^{k}}\,\frac{1}{{\left(1+r\right)}^{n-k}}\,\log\left(\frac{r^{k}}{{\left(1+r\right)}^{k}}\right)\right]-\sum_{k=0}^{n}C_{n}^{k}\,\left[\frac{r^{k}}{{\left(1+r\right)}^{k}}\,\frac{1}{{\left(1+r\right)}^{n-k}}\,\log\left(\frac{1}{{\left(1+r\right)}^{n-k}}\right)\right]$$

$$\;=-\log\left(\frac{r}{1+r}\right)\cdot \sum_{k=0}^{n}C_{n}^{k}\,\left[k\cdot \frac{r^{k}}{{\left(1+r\right)}^{k}}\,\frac{1}{{\left(1+r\right)}^{n-k}}\right]-\log\left(\frac{1}{1+r}\right)\cdot \sum_{k=0}^{n}C_{n}^{k}\,\left[k\cdot \frac{1}{{\left(1+r\right)}^{k}}\,\frac{r^{n-k}}{{\left(1+r\right)}^{n-k}}\right]$$

Assuming $p=\frac{r}{1+r}$, there is

$$\sum_{k=0}^{n}C_{n}^{k}\cdot k\cdot \frac{r^{k}}{{\left(1+r\right)}^{k}}\,\frac{1}{{\left(1+r\right)}^{n-k}}=\sum_{k=0}^{n}C_{n}^{k}\cdot k\cdot p^{k}\,{\left(1-p\right)}^{n-k}$$

which is the expression of the expectation of a binomial distribution *B*(*n*, *p*) and it equals to *np*. Thus,

$$-\log\left(\frac{r}{1+r}\right)\cdot \sum_{k=0}^{n}C_{n}^{k}\,\left[k\cdot \frac{r^{k}}{{\left(1+r\right)}^{k}}\,\frac{1}{{\left(1+r\right)}^{n-k}}\right]-\log\left(\frac{1}{1+r}\right)$$

$$\;\cdot \sum_{k=0}^{n}C_{n}^{k}\,\left[k\cdot \frac{1}{{\left(1+r\right)}^{k}}\,\frac{r^{n-k}}{{\left(1+r\right)}^{n-k}}\right]$$

$$\;=-\log\left(\frac{r}{1+r}\right)\cdot n\cdot \frac{r}{1+r}-\log\left(\frac{1}{1+r}\right)\cdot n\cdot \frac{1}{1+r}$$

$$\;=-n\,\left(\frac{r\,\log r-\left(1+r\right)\,\log\left(1+r\right)}{\left(1+r\right)}\right)$$

And the fractal dimension of blood flow for the perfect binary vascular tree with split ratio *r* is obtained as:

$$D_{Q}\,\left(r\right)=\frac{-n\,\left(\frac{r\,\log r-\left(1+r\right)\,\log\left(1+r\right)}{\left(1+r\right)}\right)}{\log2^{n}}=\frac{\left(1+r\right)\,\log\left(1+r\right)-r\,\log r}{\log2\cdot \left(1+r\right)}$$

## References

[B1] Alosco M. L. Gunstad J. Jerskey B. A. Xu X. Clark U. S. Hassenstab J. (2013). The adverse effects of reduced cerebral perfusion on cognition and brain structure in older adults with cardiovascular disease. *Brain Behav.* 3 626–636. 10.1002/brb3.171 24363966 PMC3868168

[B2] Alosco M. L. Spitznagel M. B. Cohen R. Raz N. Sweet L. H. Josephson R. (2014). Reduced cerebral perfusion predicts greater depressive symptoms and cognitive dysfunction at a 1-year follow-up in patients with heart failure. *Int. J. Geriatr. Psychiatry* 29 428–436. 10.1002/gps.4023 24022882 PMC3949179

[B3] Arciero J. C. Causin P. Malgaroli F. (2017). Mathematical methods for modeling the microcirculation. *AIMS Biophys.* 4 362–399. 10.3934/biophy.2017.3.362

[B4] Bassingthwaighte J. B. Beard D. A. Li Z. (2001). The mechanical and metabolic basis of myocardial blood flow heterogeneity. *Basic Res. Cardiol.* 96 582–594. 10.1007/s003950170010 11770077 PMC2878314

[B5] Bassingthwaighte J. B. King R. B. Roger S. A. (1989). Fractal nature of regional myocardial blood flow heterogeneity. *Circ. Res.* 65 578–590. 10.1161/01.res.65.3.578 2766485 PMC3361973

[B6] Butcher J. T. Goodwill A. G. Stanley S. C. Frisbee J. C. (2013). Blunted temporal activity of microvascular perfusion heterogeneity in metabolic syndrome: a new attractor for peripheral vascular disease? *Am. J. Physiol. Heart Circ. Physiol.* 304 H547–H558. 10.1152/ajpheart.00805.2012 23262133 PMC3566484

[B7] Cheng S. C. Huang Y. M. (2003). A novel approach to diagnose diabetes based on the fractal characteristics of retinal images. *IEEE Trans. Inf. Technol. Biomed.* 7 163–170. 10.1109/titb.2003.813792 14518729

[B8] Cheung C. Y. Tay W. T. Mitchell P. Wang J. J. Hsu W. Lee M. L. (2011). Quantitative and qualitative retinal microvascular characteristics and blood pressure. *J. Hypertens.* 29 1380–1391. 10.1097/HJH.0b013e328347266c 21558958

[B9] Chhabra A. Jensen R. V. (1989). Direct determination of the f(alpha) singularity spectrum. *Phys. Rev. Lett.* 62 1327–1330. 10.1103/PhysRevLett.62.1327 10039645

[B10] Dubin A. Henriquez E. Hernandez G. (2018). Monitoring peripheral perfusion and microcirculation. *Curr. Opin. Crit. Care* 24 173–180. 10.1097/MCC.0000000000000495 29553951

[B11] Efimova I. Y. Efimova N. Y. Triss S. V. Lishmanov Y. B. (2008). Brain perfusion and cognitive function changes in hypertensive patients. *Hypertens. Res.* 31 673–678. 10.1291/hypres.31.673 18633179

[B12] Fahraeus R. Lindqvist T. (1931). The viscosity of the blood in narrow capillary tubes. *Am. J. Physiol.* 96 562–568.

[B13] Gabrys E. Rybaczuk M. Kedzia A. (2005). Fractal models of circulatory system. Symmetrical and asymmetrical approach comparison. *Chaos Solitons Fractals* 24 707–715. 10.1016/j.chaos.2004.09.087

[B14] Gould D. J. Vadakkan T. J. Poche R. A. Dickinson M. E. (2011). Multifractal and lacunarity analysis of microvascular morphology and remodeling. *Microcirculation* 18 136–151. 10.1111/j.1549-8719.2010.00075.x 21166933 PMC3049800

[B15] Grasman J. Brascamp J. W. Van Leeuwen J. L. Van Putten B. (2003). The multifractal structure of arterial trees. *J. Theor. Biol.* 220 75–82. 10.1006/jtbi.2003.3151 12453452

[B16] Guven G. Hilty M. P. Ince C. (2020). Microcirculation: physiology, pathophysiology, and clinical application. *Blood Purif.* 49 143–150. 10.1159/000503775 31851980 PMC7114900

[B17] Haindl R. Trasischker W. Wartak A. Baumann B. Pircher M. Hitzenberger C. (2016). Total retinal blood flow measurement by three beam Doppler optical coherence tomography. *Biomed. Opt. Express* 7 287–301. 10.1364/BOE.7.000287 26977340 PMC4771449

[B18] Halley J. M. Hartley S. Kallimanis A. S. Kunin W. E. Lennon J. J. Sgardelis S. P. (2004). Uses and abuses of fractal methodology in ecology. *Ecol. Lett.* 7 254–271. 10.1111/j.1461-0248.2004.00568.x

[B19] Halsey T. C. Jensen M. H. Kadanoff L. P. Procaccia I. I. Shraiman B. I. (1986). Fractal measures and their singularities: the characterization of strange sets. *Phys. Rev. A Gen. Phys.* 33 1141–1151. 10.1103/physreva.33.1141 9896729

[B20] Hassan M. K. (2019). Is there always a conservation law behind the emergence of fractal and multifractal? *Eur. Phys. J. Spec. Top.* 228 209–232. 10.1140/epjst/e2019-800110-x 27627411

[B21] Huo Y. Kassab G. S. (2012). Intraspecific scaling laws of vascular trees. *J. R. Soc. Interface* 9 190–200. 10.1098/rsif.2011.0270 21676970 PMC3223633

[B22] Huo Y. Kassab G. S. (2016). Scaling laws of coronary circulation in health and disease. *J. Biomech.* 49 2531–2539. 10.1016/j.jbiomech.2016.01.044 26921916

[B23] Ince C. (2005). The microcirculation is the motor of sepsis. *Crit. Care* 9(Suppl. 4) S13–S19. 10.1186/cc3753 16168069 PMC3226164

[B24] Ince C. Boerma E. C. Cecconi M. De Backer D. Shapiro N. I. Duranteau J. (2018). Second consensus on the assessment of sublingual microcirculation in critically ill patients: results from a task force of the European Society of Intensive Care Medicine. *Intensive Care Med.* 44 281–299. 10.1007/s00134-018-5070-7 29411044

[B25] Katanov D. Gompper G. Fedosov D. A. (2015). Microvascular blood flow resistance: role of red blood cell migration and dispersion. *Microvasc. Res.* 99 57–66. 10.1016/j.mvr.2015.02.006 25724979

[B26] Kharche S. R. So A. Salerno F. Lee T. Y. Ellis C. Goldman D. (2018). Computational assessment of blood flow heterogeneity in peritoneal dialysis patients’ cardiac ventricles. *Front. Physiol.* 9:511. 10.3389/fphys.2018.00511 29867555 PMC5968396

[B27] Kitagawa K. Oku N. Kimura Y. Yagita Y. Sakaguchi M. Hatazawa J. (2009). Relationship between cerebral blood flow and later cognitive decline in hypertensive patients with cerebral small vessel disease. *Hypertens. Res.* 32 816–820. 10.1038/hr.2009.100 19575014

[B28] Liu H. Wang D. Leng X. Zheng D. Chen F. Wong L. K. S. (2020). State-of-the-art computational models of circle of willis with physiological applications: a review. *IEEE Access* 8 156261–156273. 10.1109/access.2020.3007737

[B29] Liu J. S. Ding W. L. Dai J. S. Zhao G. Sun Y. X. Yang H. M. (2018). Unreliable determination of fractal characteristics using the capacity dimension and a new method for computing the information dimension. *Chaos Solitons Fractals* 113 16–24. 10.1016/j.chaos.2018.05.008

[B30] Lorthois S. Cassot F. (2010). Fractal analysis of vascular networks: insights from morphogenesis. *J. Theor. Biol.* 262 614–633. 10.1016/j.jtbi.2009.10.037 19913557

[B31] Mandelbrot B. B. (1982). *The Fractal Geometry of Nature.* New York, NY: W.H. Freeman.

[B32] Muller L. O. Toro E. F. (2014). A global multiscale mathematical model for the human circulation with emphasis on the venous system. *Int. J. Numer. Method. Biomed. Eng.* 30 681–725. 10.1002/cnm.2622 24431098

[B33] Nadal J. Deverdun J. de Champfleur N. M. Carriere I. Creuzot-Garcher C. Delcourt C. (2020). Retinal vascular fractal dimension and cerebral blood flow, a pilot study. *Acta Ophthalmol. (Copenh.)* 98 E63–E71. 10.1111/aos.14232 31545560

[B34] Nayak S. R. Mishra J. Palai G. (2019). Analysing roughness of surface through fractal dimension: a review. *Image Vis. Comput.* 89 21–34. 10.1016/j.imavis.2019.06.015

[B35] Nichols W. W. Heffernan K. S. Chirinos J. A. (2015). “Overview of the normal structure and function of the macrocirculation and microcirculation,” in *Arterial Disorders: Definition, Clinical Manifestations, Mechanisms and Therapeutic Approaches*, eds Berbari A. Mancia G. (Cham: Springer International Publishing), 13–46.

[B36] Pijewska E. Sylwestrzak M. Gorczynska I. Tamborski S. Pawlak M. A. Szkulmowski M. (2020). Blood flow rate estimation in optic disc capillaries and vessels using doppler optical coherence tomography with 3D fast phase unwrapping. *Biomed. Opt. Express* 11 1336–1353. 10.1364/boe.382155 32206414 PMC7075620

[B37] Pitsianis N. Bleris G. L. Argyrakis P. (1989). Information dimension in fractal structures. *Phys. Rev. B Condens. Matter* 39 7097–7100. 10.1103/physrevb.39.7097 9947359

[B38] Pries A. R. Secomb T. W. (2005). Microvascular blood viscosity in vivo and the endothelial surface layer. *Am. J. Physiol. Heart Circ. Physiol.* 289 H2657–H2664. 10.1152/ajpheart.00297.2005 16040719

[B39] Pries A. R. Secomb T. W. (2009). Origins of heterogeneity in tissue perfusion and metabolism. *Cardiovasc. Res.* 81 328–335. 10.1093/cvr/cvn318 19028725 PMC2639106

[B40] Reglin B. Secomb T. W. Pries A. R. (2009). Structural adaptation of microvessel diameters in response to metabolic stimuli: where are the oxygen sensors? *Am. J. Physiol. Heart Circ. Physiol.* 297 H2206–H2219. 10.1152/ajpheart.00348.2009 19783778 PMC2793139

[B41] Reglin B. Secomb T. W. Pries A. R. (2017). Structural control of microvessel diameters: origins of metabolic signals. *Front. Physiol.* 8:813. 10.3389/fphys.2017.00813 29114229 PMC5660852

[B42] Schmidt A. Zidowitz S. Kriete A. Denhard T. Krass S. Peitgen H. O. (2004). A digital reference model of the human bronchial tree. *Comput. Med. Imaging Graph.* 28 203–211. 10.1016/j.compmedimag.2004.01.001 15121209

[B43] Secomb T. W. (2017). Blood flow in the microcirculation. *Annu. Rev. Fluid Mech.* 49 443–461. 10.1146/annurev-fluid-010816-060302

[B44] So G. B. So H. R. Jin G. G. (2017). Enhancement of the box-counting algorithm for fractal dimension estimation. *Pattern Recognit. Lett.* 98 53–58. 10.1016/j.patrec.2017.08.022

[B45] Stosic T. Stosic B. D. (2006). Multifractal analysis of human retinal vessels. *IEEE Trans. Med. Imaging* 25 1101–1107. 10.1109/tmi.2006.879316 16895002

[B46] Takahashi T. (2014). *Microcirculation in Fractal Branching Networks.* Tokyo: Springer.

[B47] Takahashi T. Nagaoka T. Yanagida H. Saitoh T. Kamiya A. Hein T. (2009). A mathematical model for the distribution of hemodynamic parameters in the human retinal microvascular network. *Biorheology* 23 77–86.

[B48] Van Beek J. H. Roger S. A. Bassingthwaighte J. B. (1989). Regional myocardial flow heterogeneity explained with fractal networks. *Am. J. Physiol.* 257(5 Pt 2) H1670–H1680. 10.1152/ajpheart.1989.257.5.H1670 2589520 PMC4130396

[B49] Wang R. F. Li P. L. Pan Q. Li J. K. J. Kuebler W. M. Pries A. R. (2019). Investigation into the diversity in the fractal dimensions of arterioles and venules in a microvascular network–a quantitative analysis. *Microvasc. Res.* 125:10. 10.1016/j.mvr.2019.103882 31075242

[B50] Wang Y. Lu A. Gil-Flamer J. Tan O. Izatt J. A. Huang D. (2009). Measurement of total blood flow in the normal human retina using doppler fourier-domain optical coherence tomography. *Br. J. Ophthalmol.* 93 634–637. 10.1136/bjo.2008.150276 19168468 PMC2743389

[B51] Yang J. Wang Y. (2013). Design of vascular networks: a mathematical model approach. *Int. J. Numer. Method. Biomed. Eng.* 29 515–529. 10.1002/cnm.2534 23225739

[B52] Zamir M. (2001). Fractal dimensions and multifractility in vascular branching. *J. Theor. Biol.* 212 183–190. 10.1006/jtbi.2001.2367 11531384

[B53] Zhang C. Wang L. Li X. Y. Li S. Y. Pu F. Fan Y. B. (2014). Modeling the circle of Willis to assess the effect of anatomical variations on the development of unilateral internal carotid artery stenosis. *Biomed. Mater. Eng.* 24 491–499. 10.3233/bme-130835 24211932

